# Navigating the Choice Between Wrist-Worn Research- and Consumer-Grade Wearables to Monitor Movement Behaviours: A Perspective

**DOI:** 10.3389/ijph.2026.1609200

**Published:** 2026-02-05

**Authors:** Fabian Schwendinger, Stefania Iaquinto, Valentin Jaki Waibl, Vasileios Nittas, Oliver Gruebner, Viktor von Wyl

**Affiliations:** 1 Division of Sports and Exercise Medicine, Department of Sport, Exercise and Health, University of Basel, Basel, Switzerland; 2 Division of Exercise and Environmental Physiology, Department of Sport, Exercise and Health, University of Basel, Basel, Switzerland; 3 Swiss School of Public Health, Zurich, Switzerland; 4 Department of Epidemiology, Epidemiology, Biostatistics and Prevention Institute, University of Zurich, Zürich, Switzerland; 5 Faculty of Sciences, University of Basel, Basel, Switzerland; 6 Swiss Tropical and Public Health Institute, Allschwil, Switzerland; 7 Faculty of Health Sciences and Medicine, University of Lucerne, Lucerne, Switzerland; 8 Institute for Implementation Science in Health Care, University of Zurich, Zurich, Switzerland

**Keywords:** accelerometry, equipment design, research methodology, sensor technology, wearable electronic devices

## Abstract

Wrist-worn wearables are increasingly used to monitor movement behaviours in research and daily life. While consumer- and research-grade devices share overlapping capabilities, guidance on selecting the most appropriate device for specific research contexts remains limited. We provide an expert-opinion-based perspective to support device selection based on three key dimensions: (1) contextual and procedural requirements, (2) scientific requirements, and (3) device and user requirements. The perspective is based on discussions within the Swiss School of Public Health+ ‘Big Data in Public Health’ workshop and a targeted, non-systematic literature review, as well as expert feedback. It is illustrated using two case examples: monitoring movement in Parkinson’s disease and promoting physical activity in older adults. This perspective may help novices in the field of wearable research by providing guidance that could facilitate informed decision-making, balancing scientific rigour with practical feasibility, and supporting the effective integration of wearable technologies in movement behaviour research.

## Introduction

Wrist-worn wearable technologies to monitor movement behaviours have surged in recent years, generating large amounts of data for consumers and researchers. This trend is reflected in a nearly fourfold increase in Scopus-indexed publications (2010–2020) using the terms “exercise OR physical activity” AND “accelerometer OR accelerometry,” [1]. At the same time, consumer-grade wearable use is rising; for example, in the UK, the number of users is projected to increase from 7.5 million in 2024 to 12.9 million in 2029 [2].

Broadly, wearables fall into two categories: consumer-grade devices, designed for everyday use with user-friendly features, and research-grade devices, built for scientific applications emphasising accuracy and precision. In this work, the term consumer-grade wearables primarily refers to wrist-worn devices that provide on-device user feedback (e.g., smartwatches or advanced fitness trackers), while simpler screenless consumer wearables are acknowledged but not the primary focus of this perspective. Consumer-grade wearables (e.g., Fitbit, Apple Watch) were initially tools for tracking daily activities and promoting healthy lifestyles [3]. In contrast, research-grade devices (e.g., ActiGraph, GENEActiv, Empatica) were reserved for high-quality, specialised data collection [4, 5]. Meanwhile, both are widely used in research, with consumer-grade wearables increasingly complementing or even replacing research-grade devices [6]. A growing list of both research- and consumer-grade wearables together with device specifications is available elsewhere [7].

While both wearable types present opportunities and challenges, little practical guidance exists on selecting the most appropriate device for a given research purpose. Existing studies discuss these devices’ general capabilities and measurement properties [8], but rarely address suitability for specific contexts. To address this gap, we propose an expert-opinion perspective offering a set of considerations to choose between consumer- and research-grade wearables based on specific project needs. Our focus is on wrist-worn devices and their applications in monitoring movement behaviours. We illustrated our perspective with two case examples: monitoring movement in Parkinson’s disease (PD) and promoting physical activity (PA) in elderly.

## Comparison of Research- and Consumer-Grade Wearables

**FIGURE 1:**
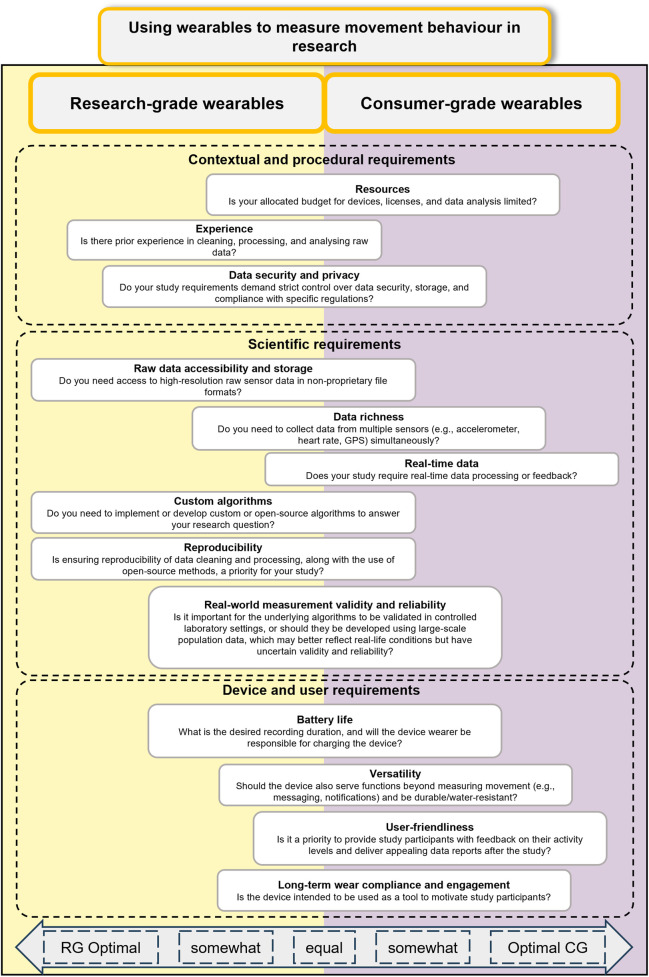
Overview of key considerations for deciding between consumer and research-grade wearables in physical activity research. The horizontal alignment of the text boxes indicates whether the respective topic is better addressed by research-grade (RG) or consumer-grade (CG) wearables if a question is answered with “Yes.” The vertical arrangement of the text boxes does not imply any prioritisation of the topics (Switzerland, 2025).

To compare research- and consumer-grade wearables, we collated a set of considerations grounded primarily in expert opinion. The initial structure emerged from discussions during the Swiss School of Public Health+ ‘Big Data in Public Health’ Workshop and a non-systematic literature review. Because our aim was to provide an overview of practical insights rather than to generate a systematic framework, this work should be understood as narrative and expertise driven. Our work is not accompanied by an evaluation. To refine the preliminary dimensions, we sought feedback from three researchers within our network who were selected based on their active engagement and publication record in wearable-based measurement research. Their input served as an informal form of peer feedback on the conceptual structure, rather than a formal, bias-controlled expert panel (see Acknowledgements). The resulting perspective (see also Figure 1) comprises three high-level dimensions: (1) contextual and procedural requirements, (2) scientific requirements, and (3) device and user requirements. These dimensions provide an overview of considerations for evaluating wearable suitability in research, balancing practical and scientific considerations.

Each dimension was divided into several sub-topics, highlighting a distinct yet interconnected aspect of wearable evaluation:

- Contextual and procedural requirements cover the practical and logistical aspects of incorporating wearables into a study, including budget constraints, data management, and alignment with study protocols for seamless integration.
- Scientific requirements encompass methodological rigour and data quality, ensuring reliable, valid, and reproducible findings. This includes data accuracy, reproducibility, and the device’s suitability for scientific analyses.
- Device and user requirements address practical and technical considerations of wearable hardware, including durability, battery life, and versatility, which influence performance and reliability in research. They also consider participants’ interaction with the device, emphasising usability, comfort, and design features that influence compliance and data quality.

### Contextual and Procedural Requirements

Resources: The cost of research- and consumer-grade wearables varies widely with features like battery life, functionality, and preferred operating system/software environment. While several consumer-grade options are available for under $200 [9], research-grade wearables may span a broader price range, with both relatively low-cost options and substantially higher-cost devices [10, 11]. Additionally, expenses for implementing software to extract raw data, post-processing, and outcome generation should be considered when selecting devices. Finally, the time required for researchers to prepare devices, extract data, and process outcomes represents an important resource consideration.

Experience: Consumer-grade wearables often rely on proprietary algorithms for data cleaning, processing, and analysis [12, 13]. This may limit researcher control. Research-grade devices, by contrast, commonly allow more independent data handling but typically demand greater data science expertise when working with publicly available algorithms like GGIR [14], biobankAccelerometerAnalysis [4, 15, 16], or FLIRT [17]. Researchers should therefore consider their available analytical expertise and infrastructure when choosing between devices, balancing ease of use against transparency, flexibility, and reproducibility.

Data security and privacy: Managing wearable data requires careful attention to security, privacy, and regulatory compliance [18]. Research-grade wearables often provide unfiltered data access, allowing researchers to independently oversee storage, processing, and analysis. Consumer-grade wearables often store data on proprietary cloud platforms, limiting such control [19]. The selection process should involve evaluating data governance policies, encryption standards, and user consent procedures to ensure compliance with ethical and legal standards while preserving data integrity and participant privacy [20].

### Scientific Requirements

Raw data accessibility: Most research-grade wearables store high-resolution raw data with full access [1]. Consumer devices often do not retain raw data to conserve battery and storage, though some allow access with prior setup (e.g., for Apple and Fitbit devices [21, 22]). Access to raw data may enable more precise adjustments and analyses tailored to research objectives. However, with consumer-grade wearables, even access to retrospective data may be possible via user consent and platform Application Programming Interfaces (APIs). This could be highly valuable for studies applying a ‘bring your own device approach’ [23].

Data richness: Consumer-grade wearables typically integrate multiple sensors, including heart rate, accelerometers, temperature, and photoplethysmography, providing a comprehensive picture of physiological state and behaviour in everyday settings. Earlier research-grade wearables were often limited to specific variables, like heart rate or acceleration, but newer models now incorporate additional sensors, aligning closer with consumer-grade devices. This advancement enables researchers to gather richer datasets from research-grade wearables while maintaining the precision and reliability required for research.

Data storage and transmission: Research-grade wearables typically prioritise local data storage and controlled data transfer. This often necessitates periodic downloads rather than continuous wireless transmission. This may limit real time processing and participant-facing feedback. Yet, emerging solutions are beginning to address these limitations. In contrast, consumer-grade wearables typically support real-time tracking and feedback to users.

Custom algorithms: Raw data access allows researchers to apply custom or open-source algorithms for data processing, tailoring analyses to specific needs. While this is more common with research-grade wearables, an increasing number of consumer-grade wearables are starting to recognise this need and offer the implementation of custom algorithms. See, for example, Creagh, Hamy [24].

Reproducibility: Open-source methodologies for data processing enhance transparency and reproducibility, a feature more common in research-grade wearables. While some consumer-grade wearables facilitate open-source methods, their metrics frequently rely on proprietary algorithms with uncertain validity and reliability. Moreover, these algorithms may change over time with limited versioning, impairing the comparability to earlier data [25].

Real-world measurement validity and reliability: The algorithms used to derive movement behaviour metrics in consumer-grade wearables are continuously evolving and based on real-life data collected from many users over extended periods. This can result in greater data variance compared to research projects with limited observation periods, but it may also allow commercial algorithms to better capture everyday behaviour. A recent meta-analysis suggests that while research-grade devices may offer advantages for total energy expenditure, consumer-grade devices can outperform research-grade monitors in domains such as ambulation and sedentary behaviour [26]. For many consumer-grade devices, the validity of derived metrics remains unclear, particularly as newer algorithm versions are introduced, and accuracy may vary substantially by device type and brand. The validity and reliability of proprietary algorithms are further complicated by the fact that updates often occur without notice. Increasingly, open-source algorithms based on large datasets from research-grade wearables are becoming available, potentially closing this gap [27]. Moreover, the need for harmonisation of data from different wearables and algorithms has been recognised. The reader is directed elsewhere for additional information [23].

### Device and User Requirements

Battery life: Runtime may vary widely across consumer- and research-grade wearables and depend on features (e.g., display, sensors), sampling rates, and power management. Many research-grade wearables can record for days to weeks without recharging but high sampling frequency shortens runtime [1]. Consumer-grade wearables range from multi-day trackers to smartwatches that need to be charged more frequently. Consequently, comparing expected runtimes for the specific study protocol is key. If participants are responsible for charging the devices, periods of non-wear may occur, potentially impacting compliance.

Versatility: Researchers or participants may seek diverse sensor data to more comprehensively describe lifestyle and physiological parameters. Research-grade wearables are designed to perform essential research functions, whereas consumer-grade wearables include additional features, like step tracking, heart rate monitoring, and receiving notifications. These broaden measurement possibilities, allow retrospective analyses of previously collected data, and may reduce the need for new equipment [28, 29]. Consumer-grade wearables are also often designed with durability and water-resistance in mind, supporting long-term use in diverse daily environments.

User-friendliness and ease of understanding: Consumer-grade wearables prioritise the end user, offering intuitive interfaces, attractive designs, seamless smart device integration, and appealing visualisations of outcome data. Research-grade wearables typically focus on data accuracy and reliability, often sacrificing user-friendly features to ensure high-quality, customisable data collection for researchers, which limits user-friendliness. However, these distinctions are increasingly narrowing as research-grade devices adopt more user-friendly designs and feedback mechanisms.

Long-term wear compliance and engagement: The capacity for self-quantification enabled by consumer-grade wearables has the potential to intrinsically motivate users to become more active. They may also serve as an incentive by offering users insights about themselves rather than merely serving as a data source for researchers. However, real-time tracking and data display can also exert pressure, potentially reducing motivation and wellbeing [30]. Many consumer-grade wearables permit users to conceal selected outcomes if desired. While research-grade wearables have traditionally lacked displays and therefore provided no feedback to participants, an increasing number of devices are now emerging that offer similar capabilities comparable to those of consumer-grade devices [31, 32].

## Practical Application of the Proposed Perspective: Examples

### Monitoring Tremor in Parkinson’s Disease (PD)

This example aims to monitor tremor progression in PD, a neurodegenerative condition affecting motor and non-motor systems, with hand tremors as a common symptom [33]. Traditional movement behaviour metrics, like step counts or activity intensity, insufficiently track tremor progression, due to their inability to measure tremor-specific characteristics like frequency, amplitude, or temporal fluctuations [34]. Instead, wrist-worn wearables are used to develop digital biomarkers for accurately tracking tremor severity over time [35].

The perspective may guide decision-making as follows:

- Contextual and procedural requirements: When budgetary limitations are considerable, and comprehensive raw data is not essential, consumer-grade wearables may be an adequate alternative. Nevertheless, research-grade wearables are optimal for those requiring high-resolution raw data access.
- Scientific requirements: Developing tremor biomarkers often requires custom algorithms and reproducible methods, favouring research-grade devices with transparent data pipelines. Consumer-grade wearables offer multi-sensor data and, in some cases, raw data access, but their reliance on proprietary algorithms makes them less reliable for capturing tremor dynamics.
- Device and user requirements: Battery life is critical for long-term monitoring. Research-grade wearables, designed primarily for data collection, typically last longer. In contrast, consumer wearables, with their additional non-essential features, tend to have shorter battery life. However, if regular recharging is acceptable, they remain a viable option.

If participants require user-friendly devices with motivating features, consumer wearables may be preferable. However, the technical demands of tremor monitoring favour research-grade wearables, especially if the researchers have experience with raw data processing.

In conclusion, research-grade wearables are generally better suited for monitoring tremor progression in PD due to their high-resolution raw data, customisable algorithms, and flexibility in handling complex symptoms. Consumer-grade wearables with raw data access can be a cost-effective alternative when budget or user-friendliness is prioritised and where limitation in algorithm transparency are acceptable. As technology evolves, more consumer-grade solutions might emerge specifically targeted to this task. Such devices might pose a user-friendly alternative that could also be valuable for research purposes.

### Promoting PA in Older Adults in Everyday Settings

This example examines an intervention study promoting PA in adults aged 65+ years in a free-living setting. Promoting PA is crucial to counteract age-related decline in PA and improve health outcomes [36]. However, this population may represent a heterogenous group with substantial variability in mobility patterns, physical function, technical proficiency, and preferences regarding data use and privacy.

The perspective may guide decision-making as follows:

• Contextual and procedural requirements: When budgets are limited and high-resolution raw data are unnecessary, consumer-grade wearables offer a cost-effective solution. However, in intervention studies where effect estimates depend on change in specific PA measures (e.g., step count), systematic measurement error like underestimation of steps due to different gait patterns or assistive device use might distort intervention effects [37]. Research-grade devices may be warranted if the study requires detailed raw data or complex post-processing and consumer-grade devices are available with demonstrated validity in the target population.
• Scientific requirements: Reproducibility may be important for an intervention study in a free-living setting, but custom algorithms are likely less critical. Consumer wearables are often sufficient for tracking overall activity levels. However, if validated activity metrics are required, consumer-grade wearables with established reliability or open-source research-grade wearables may be preferable.
• Device and user requirements: Older adults may differ widely in technical skills, visual acuity, cognitive capacity, and attitudes toward data sharing. Devices should thus be easy to operate. Consumer-grade wearables generally meet these needs and add features like reminders, goal-setting, and real-time feedback, which can support engagement and adherence. However, data protection expectations may also vary substantially and should be considered when selecting platforms reliant on proprietary cloud infrastructures.

In conclusion, consumer-grade wearables can be suitable for PA promotion interventions in older adults, particularly when engagement and scalability are primary objectives. However, their appropriateness depends on participant characteristics, outcome measures, and tolerance for measurement uncertainty. Where systematic error due to insufficient validity in the target population is likely, more differentiated device selection may be warranted. This example illustrates that this perspective is intended to support structured, context-sensitive judgement rather than prescribe a single optimal device class.

### Limitations

While this perspective may offer guidance for selecting between consumer- and research-grade wearables, it has several limitations. The proposed dimensions are broad and may not capture all context-specific factors, like rapid advances in device capabilities, evolving regulatory requirements, or population-specific considerations. Moreover, the perspective does not replace validation studies or device comparisons, which remain essential for ensuring measurement accuracy and reliability in specific research settings. The considerations presented are grounded primarily in expert opinion and informal consensus rather than systematic evidence synthesis. This work should thus be understood as narrative and expertise-driven rather than as a comprehensive or systematically derived framework. Furthermore, the perspective has not been empirically evaluated. It may therefore reflect a limited range of perspectives. Future work should focus on systematic evaluation, broader stakeholder engagement, and the development as well as evaluation of a decision framework (e.g., in the form of a decision tree or structured questionnaire that yields a recommendation based on the weighting of selected dimensions). A Delphi study might serve as a useful starting point.

### Conclusion

Choosing a wrist-worn wearable for research should begin with aligning the study’s objectives and design with the technology. Our perspective fills an important niche by outlining expert opinion and literature-based considerations, such as data precision, customisation, user needs, and budget, and by offering guidance to help researchers consider whether research-grade or consumer-grade wearables may be more suitable for a given project.
